# Stigmatization, psychological and emotional trauma among frontline health care workers treated for COVID-19 in Lagos State, Nigeria: a qualitative study

**DOI:** 10.1186/s12913-021-06835-0

**Published:** 2021-08-21

**Authors:** Ayi Vandi Kwaghe, Olayinka Stephen Ilesanmi, Peter Okpeh Amede, James Olatunde Okediran, Rowland Utulu, Muhammad Shakir Balogun

**Affiliations:** 1 Department of Veterinary and Pest Control Services, Federal Ministry of Agriculture and Rural Development, Abuja, Nigeria; 2 Nigeria Field Epidemiology and Laboratory Training Programme, Abuja, Nigeria; 3 National Tuberculosis, Leprosy and Buruli Ulcer Control Programme, Abuja, Nigeria; 4 Department of Community Medicine, College of Medicine, University of Ibadan and University College Hospital, Ibadan, Oyo Sate Nigeria; 5 Nigerian Correctional Service, Directorate of Health and Social Welfare, Bauchi, Bauchi State Nigeria; 6 Department of Public Health, Federal Capital Territory Administration, Abuja, Nigeria; 7 Nigeria Centre for Disease Control, Abuja, Nigeria; 8 Alex Ekwueme Federal University Teaching Hospital, Abakaliki, Ebonyi State Nigeria

**Keywords:** Stigmatization, Psychological trauma, Moral trauma, Isolation, Challenges, Recommendations, Lagos State

## Abstract

**Background:**

COVID-19 pandemic has resulted in global health and economic crisis. We investigated the experiences of frontline health care workers recovering from COVID-19 in Lagos State Nigeria.

**Methods:**

We conducted a qualitative study among frontline health workers recovering from COVID-19 in Lagos State, Nigeria. We interviewed 12 respondents before achieving data saturation. We used a checklist to guide the interview according to the phenomenon under study. Data obtained were analyzed using Colaizzi’s phenomenological method.

**Results:**

The study was summarized under five themes: knowledge of COVID-19, exposure, reactions, challenges and recommendations. The respondents were quite knowledgeable on COVID-19, their reactions when informed of their status were denial, anxiety, distress, disorientation, crying for fear of stigmatization, while some were psychologically prepared. Reactions from colleagues, family and friends were encouraging and provided solace for them with a few colleagues and families that had negative reactions. Challenges include anosmia, movement restriction, loneliness, worries about the state of their families, nondisclosure of status to family members, non-conducive isolation centre with limited space, insomnia, stigmatization by health workers at the isolation centre, extended duration of stay, delay in the release of test results and use of ambulance for evacuation to the isolation centres. Coping strategies were watching movies, phone calls, use of social media, listening to music, attending webinars, working on projects and reading spiritual books. Recommendations were early laboratory testing of samples and conveying of results, increase testing capacity, the need of health care workers to be more compassionate, better method of evacuation of people that tested positive to COVID-19, aside the use of ambulance that increases the likelihood of stigmatization and standard guideline for the case management of people recovering from COVID-19 in Lagos state.

**Conclusions:**

Respondents felt stigmatized and psychologically and morally traumatized. Isolation is a difficult experience and some negative emotions as expressed by previous studies were experienced by the respondents. There is need for increased testing capacity, timely results dissemination, early evacuation and creation of more isolation centres in Lagos State due to the rising number of cases and shortage of bed space.

**Supplementary Information:**

The online version contains supplementary material available at 10.1186/s12913-021-06835-0.

## Background

The World Health Organization (WHO) in January 2020 declared the outbreak of the novel Coronavirus disease (COVID-19) caused by Severe Acute Respiratory Syndrome Corona Virus 2 (SARS-CoV2) a Public Health Emergency of International Concern and a pandemic in March, 2020 [1]. China was the first country affected by the pandemic [1]. Several unique characteristics of China’s COVID-19 epidemic patterns and its management policy prompted a heightened public mental health crisis [2]. Stigma is a major challenge in the rapidly expanding global COVID-19 crisis turning out to be a long term economic and social crisis [3]. The COVID-19 pandemic is causing a lot of stigmatization because the disease is new with so many unknown factors inducing fear in people resulting in their negative reaction towards the disease [4]. In many parts of sub-Saharan Africa, there are evidence of severe stigmatization of people who have recovered and those recovering from COVID‐19 along with their families and close associates [5–7].

Frontline health care workers are people that provide direct services to their communities. Providing lifesaving care and treatment, and investments in their training and ongoing support leading to tremendous health and economic returns. Frontline health workers consist of various health workers such as nurses, midwives, community health workers, doctors, pharmacists and others that provide care directly to their communities [8]. Stigmatization of frontline health care workers in the face of the COVID-19 pandemic has been reported in different parts of the world reflecting the ordeal of these health care workers.

Quarantine and isolation measures implemented to curtail the spread of COVID-19 have affected many individuals. Quarantine is the separation and restriction of movement of people exposed to a contagious disease in order to determine their status and reduce the risk of infecting others [9] while isolation is the separation of people diagnosed with a contagious disease from people who are not sick [10]. Some negative effects of quarantine are psychological effects; post-traumatic stress symptoms, confusion, and anger [11] along with possible stigmatization from the community.

Stigma is a global barrier to health-seeking behaviours [12] and results in various forms of discrimination leading to a reduction in or lack of social acceptance or opportunities to individuals or group. Social stigma in the context of health is the negative association between a person or group of people who share certain characteristics of a specific disease [4]. In an outbreak, this may mean people are labelled, stereotyped, discriminated against, treated separately, and/or experience loss of status because of a perceived link with a disease [13]. Stigma from diverse literatures results from “misinformation, feeling of insecurity, fear of responsibility, administrative malfunction, and lack of trust on treatment; usually exhibited in the form of humor-prone stigma, residential stigma, organizational stigma, community-stigma, and apathetical stigma [14]. Furthermore, effects of stigma include; health-risks, harassment, discrimination, life-insecurity, psychological disorder, loss of social capital and emotional capital, shattering family bond and social solidarity that work as barrier to community well-being” [14]. Hence, the relevance of stigma mitigation in the face of the ongoing COVID-19 pandemic.

Stigma has a high impact on health care workers’ outcomes [15]. Stigma may influence worker compliance and can guide management communication strategies relating to pandemic risk for health care workers [15]. This study aimed at describing the experiences of frontline health care workers treated for COVID-19 infection in Lagos State Nigeria.

## Methods

### The aim, design and setting of the study

The study sites for this study are in Lagos state, southwestern part of Nigeria. One of the sites is a 50-bedded hotel which was adapted as an isolation centre for the management of COVID-19 patients. The hotel is located in Lekki, Eti-Osa local government area (LGA). The second site, Mainland Hospital, located in Yaba, Lagos Metropolis, is the first of the five COVID-19 treatment facilities in Lagos (as at time of the study). It is a 115-bedded admission facility with a female to male bed space ratio of 30:70. We conducted a qualitative study on frontline health workers receiving treatment for COVID-19 at two isolation centres in Lagos, and from a patient that is self-isolating due to lack of space at the isolation centres. This was done to report a broad range of experiences depending on where an individual was isolated. This study was also conducted to explore the issue of stigmatization based on the experiences of the frontline health care workers recovering from COVID-19.

The inclusion criteria were frontline health workers engaged in the management of COVID-19 positives either in the hospital or on the field, who were recovering from COVID-19 at the time this study was conducted. The exclusion criteria were defined as inability to participate in two or more interviews by an individual during the study period. We purposively sampled the participants in this study. We determined the number of respondents by interviewing frontline health workers that met the inclusion criteria until ‘saturation point’ was reached where no new information was generated. The interview was conducted within a period of eleven days (5th May to 15th of May, 2020) with a total of twelve participants engaged in the study. We conducted face-to-face in-depth interviews of 6 epidemiologists (4 Medical epidemiologists, 1 veterinary epidemiologist, 1 laboratory epidemiologist) a medical doctor, 2 nurses, a pharmacy technician, and 2 hospital staff hygienists). The participant on self-isolation was however interviewed over the phone. This interview method was adapted for greater privacy and confidentiality in exploring individual views and in-depth information. This method is also more suitable for sensitive issues like living with COVID-19. All COVID-19 protocols were duly observed while conducting the interviews. The respondents’ comments were transcribed verbatim by the interviewers to effectively communicate the experiences of the respondents.

As at the time of the interview, all authors as well as other field epidemiologists were residing in Lagos as part of the COVID- 19 response team. All the people that conducted the interview were frontline healthcare workers that had the permission to move around during the lockdown period. Access was obtained from the authorities at the facilities to carry out the study. A working relationship existed between some of the authors and the caregivers in their line of duty as frontline healthcare workers; frontline healthcare workers were able to influence participation to a better extent. In line with the ethical principle of voluntary participation, all patients that met the inclusion criteria but declined participation were exempted from the study. There was no relationship between the participants and the authors.

Four Epidemiologists who are also among the authors (AVK, OSI, POA and JOO), 3 males and a female served as interviewers for data collection. All interviewers had been trained in qualitative research; 3 are current students of the Nigeria Field Epidemiology and Laboratory Training Programme (NFELTP), and 1 is a graduate of NFELTP and has bagged a Master’s degree in Public Health (Field Epidemiology). COVID-19 protocols, such as physical distancing and use of face masks, were duly observed by the interviewers during the interview.

### Checklist

A checklist was developed for this study and face validity was done by a team of field epidemiologists. This was to guide the interviewers during the discussion. The checklist included questions such as: What do you understand by COVID-19? What are the ways COVID-19 could be spread? Any idea on how you contracted COVID-19? How did you feel when you were classified as a confirmed case? How did your colleagues react to your COVID-19 positive result? Did you observe any form of stigmatization by your community? How have you been coping? What are your challenges so far regarding your mental health and isolation? What are the measures that can be taken to alleviate your present negative experiences? Were you able to disclose your status to your family and friends? If yes, what was their reaction? If no, what were your reasons for not disclosing your status to your family/friends? What are your recommendations to people that are living with COVID-19? Any recommendation to improve on services rendered so far? Probing statements, such as “Please tell me more about that”, were used to enhance the depth of discussion.

### Data analysis

We conducted qualitative analysis of our data using the Colaizzi’s phenomenological method. Descriptive phenomenology reveals the “essence” or “essential structure” of a phenomenon under investigation. We collected data using explicit first person accounts experience using face-to-face and phone interviews [16]. The data set was manually coded using inductive coding method which is data-driven coding [17]. This method of coding was used to eliminate possible bias during the data analyses. Initial response and observation to construct a coding scheme based on the major categories that evolved was conducted. We conducted the analyses using the seven vital steps while sticking to the data; familiarizing ourselves with the data, identifying significant statements with direct relevance to the phenomenon under investigation, formulating meanings, clustering themes, developing exhaustive description of the phenomenon while incorporating all the themes produced, producing the fundamental structure and seeking verification of the fundamental structure by some of the participants. This method of qualitative analyses is commonly used in the health sciences [16].

## Results

The age range of the 12 health care workers interviewed was 27 to 45 years of age, of which 7 (58.3 %) were females and 5 (41.7 %) were males (Table 1). These health care workers had been involved in the management of COVID-19 positive cases either in health facilities or on the field prior to their commencement of self-isolation after testing positive for COVID-19.

**Table 1:** Characteristic of respondents

S/No	Sex	Occupation	Participant Identification
1	Female	Epidemiologist	E1
2	Male	Epidemiologist	E2
3	Male	Epidemiologist	E3
4	Male	Epidemiologist	E4
5	Male	Epidemiologist	E5
6	Female	Epidemiologist	E6
7	Female	Medical Doctor	MD
8	Female	Nurse	N1
9	Female	Nurse	N2
10	Female	Pharmacy Technician	PT
11	Female	Hygienist	H1
12	Male	Hygienist	H2

### Theme: knowledge on COVID-19 by respondents

All the respondents (*n* = 12) had knowledge of the virus while two (*n* = 2) of the 12 respondents stated that transmission could be air-borne due the high rate of infectivity of the virus. Codes on knowledge of COVID-19 by respondents and the frequency of codes mentioned by respondents indicated in Table 2.

**Table 2:** Themes, subthemes and codes obtained from data analysis

Theme	Subtheme	Codes	Frequency of codes mentioned by participants
**Knowledge on COVID-19**	Knowledge of the virus	Causative agent	(E1)*1/(E2)*1/(E3)*1/(E4)*1/(MD)*1/(N2)*1/(H2)*1
		Pathophysiology	(E1)*3/(E3)*1
		Clinical signs	(E1)*9/(E3)*3
	Mode of transmission	Mode of transmission	(E1)*2/(E2)*2/(E3)*1/(E4)*2/(MD)*2/(N1)*1/(N2)*1/(PT)*1/(H1)*1
	Prevention and control	Prevention and control	(E1)*6/(E2)*7/(MD)*5/(H2)*2
**Exposure**	likely means of exposure	Place of work	(E1)*1/(E2)*1/(E3)*1/(E4)*1/(E5)*1/(E6)*1/(MD)*1/(N1)*1/(N2)*1/(PT)*1/(H1)*1/(H2)*1
**Reactions**	Immediate reactions form positive patients	denial due to being asymptomatic	(E1)*1/(MD)*1/(H1)*1
		Feeling disturbed	(E3)*1/(E6)*2/(N1)*2/(N2)*3/(H1)*1/(H2)*2
		Felt comforted	(E2)*2/(E5)*3/(N2)*1/(H2)*1
		Delay in receiving result	(PT)*2/(H2)*1
**Reactions**	Reaction from colleagues	Supportive responses	(E1)*2/(E2)*5/(E3)*4/(E4)*3/(E5)*6/(E6)*4/(N2)*1
		No reactions	(MD)*1/(N1)*1
		Felt stigmatized	(E2)*1/(PT)*2/(H1)*2/(H2)*1
	Reaction of the family and friends	Family members not informed	(E2)*1/(E3)*1/(E4)*1/(E5)*1/(E6)*1/(PT)*1
		Reasons for not informing family members	(E2)*4/(E3)*1/(E4)*1/(E5)*1/(E6)*3/(PT)*1
		Informed friends	(E1)*1/(E4)*1
		Informed family	(E1)*1/(MD)*1/(N1)*1/(N2)*1/(H1)*1/(H2)*1
		positive response from family	(E1)*2/(N1)*4/(H1)*3/(H2)*2
		Negative response from family	(MD)*4/(N2)*3
	Reaction by community	Informed hotel management	(E1)*1/(E2)*1
		Community not aware	(E1)*1/(E2)*1/(E3)*1/(E4)*1/(E5)*1/(E6)*1/(MD)*1/(N1)*1/(N2)*1/(PT)*1/(H1)*1/(H2)*1
**Challenges and coping strategies**	Challenges	Late processing of samples	(E4)*1/(E6)*3/(H1)*1
		Movement restriction	(E2)*2/(E3)*1/(E5)*1/(E6)*1/(PT)*1/(H1)*1/(H2)*1
		Stigmatization	(E1)*3/(E4)*1/(E5)*1/(MD)*1/(N1)*1
		Hardly sleep	(E4)*1/(H2)*1
		Unconducive isolation tent	(E4)*4
		Separation from family	(E1)*1/(PT)*1/(H1)*2/(H2)*1
		Reaction to medication	(E1)*1/(E5)*1
		Late meals	(E5)*1/(H2)*1
		Development of symptom	(E3)*1
	Coping strategies	Webinar, reading and working on projects	(E2)*2/(E4)*1/(N1)*1
		Watching movies	(E4)*2/(E6)*1
		Listening to music	(N1)*1
		Phone calls	(E4)*1/(E6)*1/N1*1
**Recommendations**	Recommendation for positive patients	Advice and encouragement	(E1)*2/(E4)*1/(D1)*2/(N2)*3/(H2)*1
	Recommendations to improve outbreak response and case management	Required empathy from case management team	(E1)*1/(E5)*1/(MD)*1/(N1)*1/(PT)*1
		Timely results and evacuation	(E3)*1/(E5)*1/(MD)*3/(N2)*2/(H1)*3
		Treatment policy	(E4)*1/(E5)*1/(MD)*1
		Change evacuation process	(N1)*1/(N2)*1
		Reduce overcrowding	(E4)*1
		Public awareness Timely meals	(H2)*1 (E5)*1/(H2)*1

#### Subtheme: knowledge of the virus

Seven of the participants (*n* = 7) mentioned the causative agent as SARS COV2 or the disease is caused by a virus while two participants talked on the pathophysiology and clinical signs of the disease (Table 2). Some of the respondents’ knowledge is narrated as follows.

*“It is a viral infection, affects the respiratory system and other systems of the body, that may result in multiple organ failure. Symptoms include difficulty in breathing, runny nose, diarrhea, chest pain, headache, fever, muscle pain, weakness, arthritis and in some cases, the individual may be asymptomatic. The disease is more severe in people that are advanced in years, about 20 % case fatality in people above the age of 75 years” (E1)*.

*“COVID-19 is a viral disease, affects the respiratory system and is caused by corona virus specifically SARS-COV-2. Transmitted via droplets usually presents with fever, cough and respiratory distress” (E3)*.

*“Is a viral disease caused by SARS-CoV-2 that is highly infectious, spread from person-to-person through droplets from respiratory tracts of infected persons to their close contacts when they cough, sneeze or shout and can also be transmitted when someone comes in contact with contaminated surfaces and/or fomites and touches his nose, eyes or mouth. It can also be transmitted via aerosols but airborne transmission has not been reported” (E4).*

#### Subtheme: mode of transmission

The frequency of codes on the mode of transmission of COVID-19 is shown in Table 2. Some of the participants narrated the mode of transmission of COVID-19 as follows.

*“COVID-19 is a viral disease that is transmitted through droplets from the mouth, and nose. Transmission can occur through direct contact of droplets with the eyes, nose and mouth or indirectly by touching surfaces contaminated with droplets. The hand serves as a means by which the droplets are carried to from contaminated surfaces to the mouth, nose and eyes” (E2).*

*“A viral infection transmitted via aerosol, droplets or contaminated surfaces by touching the face with contaminated hands” (MD).*

*“Viral infection, transmitted through droplets from coughing, sneezing and touching surfaces” (N2).*

#### Subtheme: prevention/control

Four respondents’ (*n* = 4) talked on the measures of prevention and control of C0VID-19 (Table 2). Some of their narrations is as follows.

*“Preventive measures include Social distancing (2 meters), use of hand sanitizers (70 % alcohol) to disinfect the hand, proper washing of the hand under running water for about 30 seconds, cleaning of high touched surfaces with disinfectant, the use of face mask and staying away from high risk areas” (E1).*

*“The spread can be prevented or reduced by ensuring strict environmental hygiene, regular washing of hands with soap and running water for at least 20 seconds or the use of hand sanitizers with at least 60 % alcohol, cough into flexed elbow or a tissue and immediately disposed hygienically, keep at least 2 meters’ physical distance and avoid crowded environment and the use of face mask” (E2).*

#### Theme: likely means of exposure

Eleven respondents reported that they had primary contact with confirmed case or cases (*n* = 11) while one was a secondary contact of a confirmed case. All the participants (*n* = 12) likely place of exposure was at workplaces (hospitals/clinics) or at their various duties on the field during the outbreak investigation and mitigation (Table 2). This was because a number of their colleagues tested positive and they had contacts with them before they went for testing. A respondent was not sure if the infection was acquired at the place of work. Some of the participants’ experiences is narrated as follows.

*“I must have been exposed through infected friends at my place of work. Some of my colleagues were infected and as contacts I went for my test and it was positive” (E2)*.

*“The infection was contracted through colleagues at the treatment centre and my screening test came out positive” (E3).*

*“I think I was exposed at my place of work. Some of my close colleagues tested positive to the disease warranting me to also check my status and it turned out positive” (E5).*

*“I really don’t know, I am a frontline health worker and in the case investigation team and my work involves filling of case investigation forms for suspect cases, so, I wouldn’t know if some are positive cases” (E6).*

***“*** *I can’t really tell but I came in contact with a staff of the hospital that later on became positive. The staff tested positive after seven days of my consultation. A lot of people tested positive in my hospital. I had no symptoms, I went and tested because my contact became positive” (MD).*

*“We managed a positive patient at work. I wasn’t the primary person that managed but one of my colleagues” (N2)*.

### Theme: reactions

#### Subtheme: immediate reactions from positive patients

Reaction of positive frontline health workers on receiving the news includes initial denial due to being asymptomatic (*n* = 3), feeling disturbed (*n* = 6), anxiety, crying; some for fear of stigmatization, weakness, distress, pain, feeling disoriented, none specific reaction because the patients mind was prepared by a superior officer, looking forward for evacuation due to stigmatization by family members (Table 2). Some of the participants (*n* = 2) were apprehensive due to the delay in receiving their test results (Table 2).

Other positive reactions despite tested positive were faith in God, bracing up to face the worst outcome after taking the test, overcoming the shock phase before the result was out, consoled by the recovery rate despite the test outcome, positivity that they will be negative in a few days, some felt that the situation could be avoided if only they wore mask while attending meetings and the issue of death did not cross their mind at any point. The experiences of some respondents is narrated as follows.

*— “I did not feel bad I knew that I would be negative within a few days to week. Though, I felt I could have avoided being positive. This would have been accomplished by using face mask while attending meetings. I was not symptomatic, and I saw that as a good prognosis” (E2).*

*— “Though felt bad initially because I thought I had been careful enough not to get infected but knew it’s just a matter of time that I will be let out of the treatment centre” (E3).*

*— “Because of the delay in results and the symptoms I already had, I was already expecting to be positive” (E4).*

*— “I had encouraged myself and brace up for whatever outcome. So when I was called that I tested positive, I had already gone through that phase of shock, I took it in good fate and looked forward to be evacuated” (E5).*

*— “Initial denial because I don’t have any symptom. I was already looking forward for evacuation because it was delayed” (MD).*

*— “I felt bad but I was consoled that the recovery rate is high. I also had it before my grand mum. I felt I infected her and I felt very bad about that. I cried the first few days. Death did not cross my mind at any point” (N2).*

*— “I felt Weak, I comforted myself on finding out that I was not the only positive case in the facility and putting my faith in God” (H2).*

#### Subtheme: reaction from colleagues

Majority of the respondents’ colleagues (*n* = 7) were empathic, supportive, always called and encouraged the patients to stay positive, prayed fervently for the patients, assured patients that they will come out victorious and came visiting with provisions (Table 2). On the contrary, some respondents (*n* = 4) had negative reactions from their colleagues, they were stigmatized by their colleagues (Table 2). Two respondents (*n* = 2) had no reaction from colleagues because they were at home when their results came out (Table 2). The experiences of respondents were narrated as follows.

*— “Most of my colleagues were supportive, they came visiting and came with gifts. Only a few behaved as if it could not have been them. My immediate supervisor called to encourage and counselled me. He is a consultant Psychiatrist; he did a good job on me. He also shared my contact with another Psychiatrist who called and encouraged me as well” (E2).*

*— “I had very good support from colleagues both clinical and non-clinical which was encouraging. Calls and visits regularly was strengthening. Non-specific untoward reaction was noticed. Senior colleagues who had the same experience called to encourage me and also close friends who were not at my present location” (E3).*

*— “They were quite supportive. They called frequently, prayed fervently for me, ensured me that I will come out victorious and some came visiting with goods. My team lead immediately started crying when I informed her of my positive status. Both my team and Pillar leads were highly supportive” (E5).*

*— “they were sympathetic and they shared in my fears and tried to allay my fears and told me that I am fine especially the fact that I am not symptomatic” (E6).*

*— “None specific, though my boss had prepped my mind already before telling me the result of the test” (N1).*

*—* ***“*** *Some of my colleagues reacted in not too good manner and one of them promised never to eat food procured by me again” (PT).*

*— “Stigmatized by colleagues, a colleague of mine said that I was the one that brought the disease to them, that I use to bring some Hausa people into the health facility” (H1).*

#### Subtheme: reaction of the family and friends

Six (*n* = 6) of the respondents disclosed to their family and friends about their disease status while the rest did not disclose their status. Two of the respondents that disclosed their status had negative response from family members while the rest (*n* = 4) revealed that they got maximum support from their family and friends which turned out to be helpful emotionally and psychologically. They were prayed for, encouraged, and visited by family members and friends (Table 2). The rest of the respondents (*n* = 6) kept their status away from their family members for fear of inflicting distress, worries and possible psychological breakdown (Table 2). Respondents narrated their experiences in this regard as follows.

*“Disclosed it to my sister in-law and my friend. They were really empathic and supported me throughout my period of stay” (E1).*

*“I could not inform my immediate family members; my wife will be devastated. I want to prevent the trauma. She will become anxious to see me and it will be difficult for her to travel to Lagos” (E2).*

*“I could not inform my wife till now for fear of her not taking the news well. I informed my brother who is a medic too and he has been supportive” (E3).*

*“I stay with my uncle and his family, their immediate reaction was dramatic, I did expect the reaction but I was not ready for it. I was happy coming to the isolation centre because of the reaction of my family members” (MD).*

*“I live with my parents, my mom felt awfully bad, my dad felt bad, but he denied that I can’t be positive” (N2).*

*“My husband has been very supportive though I did not inform my children so that they will not be worried” (H1)*.

#### Subtheme: reaction by community

The community was oblivious of the fact that these patients were positive (*n* = 12), partly because some of them were evacuated from the hospitals where they worked and those staying in the hotels informed the authorities of their status for their rooms and the environment to be decontaminated after departure to the isolation Centre (Table 2). Some of the respondents’ experiences were narrated as follows.

***“*** *Just told the hotel people to disinfect my room while living. People around didn’t know my status” (E1).*

*“I informed the manager of the hotel where I stay, she prayed that I get well soon, I taught the manager how to decontaminate my room and she was thankful” (E2).*

***“*** *People in the community were not aware of my status” (H2).*

### Theme: challenges and coping methods

#### Subtheme: challenges

Challenges mentioned were late processing of samples (*n* = 3), movement restriction (*n* = 7), missing family (*n* = 2), anxiety and worries (*n* = 4); worries about the welfare of their families at home, long duration of stay at the isolation centre for about two weeks and are yet to be discharged, late meals (*n* = 2), insomnia (*n* = 2) and not satisfied with the psychosocial group counselling (Table 2). Other challenges adverse side effects of drugs administered (*n* = 3); some adapted to it after some days while others could not, keeping their status secret from family members because they didn’t want them to be worried, stigmatization (*n* = 5) (Table 2) by the health workers at the isolation centre; distancing themselves from the patients while wearing complete PPE. Some participants narrated their experiences as follows.

*“The workers at the hotel seem to be distancing themselves from us even while wearing complete PPE; while mopping my room, I was told to stay inside the bathroom which I did until the person finished the mopping; being shouted at because I mistakenly shut my door; away from home and alone. When I started taking the medications, I started purging profusely and I had to stop taking the medication and the purging stopped”* *(E1).*

*“Not been able to move freely as I desire. A bit of worry when I noticed a symptom that was not present before; loss of smell” (E3).*

*“The isolation centre was not conducive, not enough space in the tent, didn’t have enough sleep, noise from television from other patients” (E4).*

*“I am not finding the isolation easy, especially the fact that my result is still pending, there is no feedback from the laboratory and I have spent 16 days already in isolation waiting for my second result. have been watching movies, in and out of sleep and I have been talking with family and friends” (E6)*.

*“Some of the staff here are not empathetic, the psychosocial group counselling made me more anxious instead of me calming down” (MD)*.

*“Loneliness, worried about my children at home, staying because it is compulsory, late processing of my samples, my result came out about two weeks after taking the test and I have stayed for about 2 weeks already at the isolation centre” (H1)* *.*

#### Subtheme: coping strategies

Coping mechanisms adopted by participants during the period of isolation were making phone calls (*n* = 3), surfing the web, working on some initial projects, attending webinars and working from the isolation centre (*n* = 3), listening to music, reading spiritual books and watching movies (*n* = 2) (Table 2). Some of the participants narrated their experiences as follows.

*“Not been able to move freely as I desire, I could not do my usual work. However, I spent the time attending webinars and working from my isolation room” (E2).*

*“I copped through watching movies, having long calls with family members, working on my laptop on some of the works that I started” (E4).*

*“No challenges personally, I coped through using my phone, listening to music and reading spiritual books” (N1)* *.*

### Theme: recommendations

#### Subtheme: recommendation for positive patients

Recommendations for people recovering from COVID-19 by some of the respondents (*n* = 5) (Table 2) during the period of isolation is narrated as follows.

*“They should know that COVID-19 is not a death sentence” (E4).*

*“They should surround themselves with things that make them happy, they shouldn’t feel guilty for being positive” (D1).*

*“Those that tested positive should be optimistic while staying in isolation centre, it is not forever, they should not think of stigmatization but to survive first” (N2).*

*“They should put their trust in God” (H2).*

#### Subtheme: recommendations to improve the outbreak response and case management

To improve on the outbreak response and case management, suggestions made were; required empathy from the case management team (*n* = 5), timely presentation of results and immediate evacuation of positive patients (*n* = 5), a single guideline and treatment policy for all the isolation centres nationwide (*n* = 3) and reduction of overcrowding (*n* = 1) (Table 2). Narrations on recommendations made by some of the respondents is as follows.

*“There is need for a counsellor at the isolation centre. The person will screen and identify those who need psychological support and provide such. The Laboratory turn over time should be explained to patients and they should be informed about their progress in treatment as early as possible. Training and re training of isolation centre staff on how to handle cases is important and that been positive is not a gloomy situation” (E3).*

*“Support the government and Nigeria Centre for Disease Control by adhering strictly to all the preventive measures to avoid transmission. Special treatment for all the frontline health workers. Reduce the number of patients per tent” (E4).*

*“Regularize mealtime. Case management team should show more care to patients. Results of samples collected should be communicated not more than 48 hours after collection. There should be a single treatment policy in all the isolation centers” (E5).*

*“The staff in this centre should be more empathetic to patients, there should be standard guidelines for procedures, timely processing of samples collected, timely dissemination of results and timely evacuation of positive persons.” (MD).*

*“There should be plan for discharge after admission here, the use of ambulance should be discouraged for both evacuation and return home after treatment. People should be alerted about their status on time and not been worried for several days of weeks before result notification. Decontamination of the items owned by the persons discharged should be done in their rooms and not outside” (N2).*

*“People should be alerted about their status on time and not being worried for several days or weeks before result notification. Stigmatization by isolation centre staff should be reduced as much as possible. Health facilities should be equipped more to tackle the disease. The government should make house to house screening of population mandatory” (PT).*

## Discussion

The respondents were knowledgeable about COVID-19 which could be associated with their professional background and being COVID-19 frontline health care workers. This study revealed initial reactions to the news of testing positive to COVID-19 was denial, anxiety, fear, distress, disorientation, worries and challenges such as stigmatization, movement restriction, insomnia, loss of smell (anosmia) and unconducive environment which are similar to other qualitative studies [18–23]. Qualitative studies also identified a range of psychological responses to quarantine- such as, confusion, numbness, fear, grief and anxiety-induced insomnia [20, 24–26]. One can relate to the confusion, anxiety, and fear among the public due to the increasing incidence and mortality associated with COVID-19. Unfortunately, these factors are also fueling harmful stereotypes. This can undermine social cohesion resulting in social isolation of groups as a result of stigma [4].The impact of stigma results in difficulty in handling more severe health problems and difficulty in handling the disease outbreak. Stigma is a driving force in concealing illness to avoid unfair judgement by the society and stops individuals from seeking health care immediately and discourage imbibing healthy measures [4, 25].

The mitigation of stigma in the current COVID-19 pandemic should be geared towards addressing; enacted stigma as a result of acts of discrimination and mistreatment, felt-normative stigma hatched from community norms and values that are self-degrading and self-limiting, internalized stigma which arises from the way people view negative perspectives toward the assembly or assemblies they may belong to, and anticipated stigma that creates fear of the unknown based on worries that a person will face discrimination and prejudice in the future [27]. There is need to build on Knowledge, creating awareness, demonstrating care to people living with COVID-19 and empathizing with those affected by healthcare workers and the society at large. This will give hope to people recovering from COVID-19 infection and help them fight the infection with courage and boldness [28]. Also, we need to create a conducive environment where COVID-19 infection and its’ impact could be addressed freely with judicious actions to alleviate the spread of infection and build on the existing structure [28]. The mitigation of stigma could be inculcated through the use of media by giving audience to those that were affected by COVID-19; people who have recovered from the infection, people recovering from the infection, families of those who have succumbed to the disease and have been subjected to societal stigmatization and the frontline health care workers [28]. Bringing up pressing issues on COVI-19 based on their personal experiences on stigmatization and other issues will help in formulating public health strategies that will curb the spread of stigma in the crisis. Exploring the experiences of people who have recovered from the infection will likely induce hope in the society and may cause those that are already battling with symptoms in the society to come out for testing and accord them strength to handle stigma [28]. Drastically addressing stigma in order to minimize and possibly curb its spread in the ongoing COVID-19 pandemic is an all-encompassing responsibility, this will include the government, frontline healthcare workers, stakeholders, the media and the society, this holistic approach will ensure effective and timely mitigation of stigma [28]. Fighting stigma should be deliberate and communicated on the social media and other available media platforms based on a well thought-out plan showing supportive behaviors to people recovering from COVID-19 [4].

The unpleasantness of quarantine inculcates movement restriction, people being physically separated from their families and loved ones, uncertainty of disease status which serves as a means of creating anxiety, boredom created by redundancy which can collectively result to suicide [29]. This study relates similar findings such as movement restriction, boredom, insomnia, separation from family members and the inability to handle their day-to-day activities as part of the challenges they experienced during their period of isolation. A study of quarantined (9 days) hospital staff who might have contracted Severe Acute Respiratory Syndrome reports that quarantine is linked with symptoms of acute stress, disorder, being more prone to exhaustion, detachment from others, anxiety when dealing with febrile patients, irritability, insomnia, poor concentration and indecisiveness, deteriorating work performance, and reluctance to work or consideration of resignation [30]. In another study involving hospital staff who were examined for 3 years after quarantine experience indicated that 9 % (48 of 549) of the sample had high depressive symptoms and about 60 % (29 of 48) were previously quarantined while 15 % (63 of 424) of the group with low depressive symptoms had been quarantined relating dangers associated with quarantine [31].

This study revealed that a respondent complied to the isolation because it was compulsory. Individuals quarantined/isolated need comprehensive understanding on reasons they have to be quarantined through effective communication. Severe effects of quarantine/isolation are mostly exhibited by individuals who have been forcefully quarantined. Creating understanding on the relevance of isolation to people recovering from infection will result in less distress and reduce long-term complications when isolation is done voluntary. Public health officials need to explicitly state and explain the relevance of voluntary self-isolation and the isolation span should be short and unaltered unless in extremely relevant conditions [11, 32]. This is paramount in securing a healthy society during and after the COVID-19 pandemic.

Recommendations made to people that tested positive to COVID-19 were to put their faith in God, stay optimistic, think of themselves first irrespective of stigmatization and be conscious of the fact that testing positive to COVID-19 is not a death sentence. This can be achieved through proper counselling and educating of people recovering from COVID-19 at the various isolation centres.

Other recommendations made on improving the outbreak response were implementing standard guidelines for people at the isolation centres. Increase testing capacity, prompt processing of samples and communication of results within 48 h of sample collection. This is necessary to eliminate long waiting and long period of anxiety while waiting for the results. Furthermore, these measures will limit the spread of the virus by those people that tested positive. This is in line with the need of immediate evacuation of people that tested positive to COVID-19. Delay in the evacuation also serves as means of further spread of the disease especially for patients that can’t self-isolate due to lack of enough rooms in their houses.

There is need for health care staff at the isolation centres to be empathic with people recovering from COVI9-19 which can help in the psychological healing process. The effect of isolation especially when imposed on a person results in psychological and moral trauma, coupled with the side effects of the drug which some of them had to manage. Hence, the respondents advocated for training and retraining of staff at the isolation centres on how to interact with people recovering from COVID-19 at the isolation centres to improve on current situations. The need for physically present counsellor to improve the psychological and emotional well-being of people that tested positive at the various isolation centres who are in need of such services at the centres was also advocated for.

A respondent complained about unconducive isolation tent; too many people and noise from television resulting in his inability to rest well. People recovering form COVID-19 need to be provided with adequate isolation space and a conducive environment in order to curb impediments to testing and reporting of COVID-19 infections [33]. An isolated individual that feels suffocated by the isolation situation is likely not to comply to stay for the stipulated period and is likely to discourage others from going through the same experience [33]. The hospital management needs to address the issue of overcrowding in handling COVID-19 patients. Individuals that tested positive to COVID-19 and can self-isolate at home should be allowed to do so since there is consistent increase in the number of cases in Lagos State as the epicentre of the disease and the mounting pressure on bed space for isolation. This will alleviate the problem of bed space in the face of the pandemic. The study also revealed the need for the creation of more isolation centres in the state due to the rising demand.

Limitations of this study included the inability to conduct this study in all the isolation centers in Lagos state at the time the study was conducted. Also, differences in the experiences of frontline health care workers in other states of Nigeria who had tested positive for COVID-19 could exist. Despite these limitations, this study provides evidence to the mental effects of COVID-19 on health care workers in Lagos, the epi centre for the COVID-19 outbreak in Nigeria.

## Conclusions

The study has revealed stigmatization, psychological and emotional trauma frontline health care workers experienced while isolating during their recovery period from COVID-19. Their reactions were similar with other qualitative studies. Recommendations were also made by respondents to improve on the current existing system on outbreak investigation and management of people recovering from COVID-19 infection at the isolation centres. It is hoped that these recommendations will be acted upon by the relevant authorities for better output in the face of the pandemic.

## Supplementary Information

**Additional file 1. **Checklist

## Data Availability

The datasets used and/or analysed during the current study available from the corresponding author on reasonable request.
