# Supplementary material for: Stigmatization, psychological and emotional trauma among frontline health care workers treated for COVID-19 in Lagos State, Nigeria: a qualitative study
Source: BMC Health Serv Res. 2021 Aug 21;21:855. doi: 10.1186/s12913-021-06835-0 (PMC8380097; doi:10.1186/s12913-021-06835-0)
Supplement: Supplementary file 1 — Additional file 1. Checklist [file 12913_2021_6835_MOESM1_ESM.docx]

**Checklist**

A checklist was developed for this study and face validity was done by a team of field epidemiologists. This was to guide the interviewers during the discussion. The checklist included questions such as:

What do you understand by COVID-19?

What are the ways COVID-19 could be spread?

Any idea on how you contracted COVID-19?

How did you feel when you were classified as a confirmed case?

How did your colleagues react to your COVID-19 positive result?

Did you observe any form of stigmatization by your community?

How have you been coping?

What are your challenges so far regarding your mental health and isolation?

What are the measures that can be taken to alleviate your present negative experiences?

Were you able to disclose your status to your family and friends?

If yes, what was their reaction?

If no, what were your reasons for not disclosing your status to your family/friends?

What are your recommendations to people that are living with COVID-19?

Any recommendation to improve on services rendered so far?
